# Proposal of a domain model for 3D representation of buildings in Ecuador.

**DOI:** 10.12688/f1000research.146267.1

**Published:** 2024-04-19

**Authors:** Luis Alejandro Velastegui Cáceres, Julia Desirée Velastegui Cáceres, Pedro A Carretero Poblete, Manuel Fabian Moyón Gusqui, María Alejandra Cevallos Díaz

**Affiliations:** 1 Facultad de Ingeniería, Universidad Nacional de Chimborazo, Riobamba, Chimborazo Province, 060150, Ecuador; 2 Universidad de las Fuerzas Armadas (ESPE), Sangolqui, Pichincha, Ecuador; 3 Cuerpo de Ingenieros del Ejército, Quito, Pichincha, Ecuador

**Keywords:** Building 3D representation; infrastructure; urban development; 3D cadastre, Ecuador.

## Abstract

The accelerated urban sprawl of cities around the world presents major challenges for urban planning and land resource management. In this context, it is crucial to have a detailed 3D representation of buildings enriched with accurate alphanumeric information. A distinctive aspect of this proposal is its specific focus on the spatial unit corresponding to buildings. In order to propose a domain model for the 3D representation of buildings, the national standard of Ecuador and the international standard (ISO 19152:2012 LADM) were considered. The proposal includes a detailed specification of attributes, both for the general subclass of buildings and for their infrastructure. The application of the domain model proposal was crucial in a study area located in the Riobamba canton, due to the characteristics of the buildings in that area. For this purpose, a geodatabase was created in pgAdmin4 with official information, taking into account the structure of the proposed model and linking it with geospatial data for an adequate management and 3D representation of the buildings in an open-source Geographic Information System. This application improves cadastral management in the study region and has wider implications. This model is intended to serve as a benchmark for other countries facing similar challenges in cadastral management and 3D representation of buildings, promote efficient urban development and contribute to global sustainable development.

## 1. Introduction

Due to population growth in urban areas, cities have expanded at an accelerated rate, which in a certain way requires adequate territorial planning, for which it is necessary to have information and representation of buildings with a high level of detail to optimize decision making. The limitation of some developing countries is the lack of updated, standardized and open access cadastral information on parcels and buildings, which makes it difficult to access and integrate the information for different purposes.

In developing countries, 2D cadastral information is the most demanded, however, due to urban growth, the information provided by traditional 2D models is becoming insufficient in urban areas ( Drobež, *et al*., 2017). It should be noted that the representation of the parcel and buildings must be integrated to the alphanumeric information comprising the cadastre so that it is not considered only as a representation of the cadastral object (parcel/building) but is useful for various purposes in the area. Therefore, the implementation of 3D representation of cadastral objects (buildings) has become a necessity to solve the limitations. With this, cadastral objects are represented both at 2D and 3D level, where the information of both dimensions is integrated and represented in the same territorial management system.

That is why the 3D representation of buildings is very useful in various areas such as land planning and management, urban development, natural resources and environmental management, risk management, among others, as it provides more accurate, realistic and detailed information, which allows identifying the cadastral object with their respective public open spaces, infrastructure and urban systems in a more realistic way ( Ying, *et al.*, 2014; Kumar, Rahman & Buyuksalih, 2017; Toschi, Nocerino & Remondino, 2017a; Toschi, *et al*., 2017b).

According to Koeva and Elberink (2016) 3D physical cadastral objects refer to the buildings located on the parcel, buildings and subway constructions. Although cadastral objects are shaped in this way, it is also necessary to consider that they have a legal component and a physical component. When considered as a legal object it refers to a cadastral data model such as those proposed in ISO 19152:2012 LADM ( ISO, 2012) where rights, responsibilities and restrictions are specified. On the other hand, when considered as a physical object, it specifies the building characteristics, such as construction components, dimensions, utility, among others, which allow the representation of the cadastral object in 3D ( Aien, 2013). Integrating the two components of cadastral objects makes the 3D cadastre very useful in the multipurpose environment.

The implementation of a 3D cadastre is not a simple task, as it involves numerous challenges as demonstrated by the researches implemented so far ( Ying, *et al*., 2014; Kumar, Rahman & Buyuksalih, 2017; Toschi, *et al*., 2017a; Karki, 2013; van Oosterom, 2013; Gruber, Riecken & Seifert, 2014; Bydłosz & Bieda, 2020; Döner & Şirin, 2020; Velastegui, Rodríguez & Padilla, 2020; Ying *et al.*, 2021a, 2021b; Paasch & Paulsson, 2021; Ghawana, Janečka & Zlatanova, 2021). Different factors must be taken into account, such as: existing 2D cadastral information, technological and human resources, costs, among others. Therefore, it is important to have a physical 3D cadastral model that is the basis for implementing the 3D representation of the cadastral object (buildings) with alphanumeric information in which the most essential components are integrated and with this, in the future, the level of detail of the information can be improved to allow a geometric representation of the cadastral objects based on the physical characteristics of the buildings.

Ecuador has a 2D level cadastre that still needs to be improved and it can be clearly evidenced by the existence of a small percentage whose development is minimal in view of the lack of georeferenced data, as mentioned by Todorovski *et al*. (2018). On the other hand, taking into consideration the different levels of cadastral development established in the analysis carried out by Velastegui *et al*. (2020), it is mentioned that the tendency of most of the cantons presents an intermediate development, which implies an advanced 2D cadastre, since both geospatial and alphanumeric information have a cadastral identifier that allows the linking of both types of information and in turn both are up to the floor level, which is the minimum unit in a building block.

By having a previous analysis of the cadastral situation in Ecuador, it allows to have a perspective of the strengths and weaknesses of the current information and representation of buildings, which helps to propose the most appropriate domain model for the 3D representation of buildings and thus allows a path towards the 3D cadastre. It should be noted that the physical 3D model considers the limitations of the study area in order to be replicated in areas with similar characteristics to Ecuador.

It is important to note that municipalities update information, not only at the parcel level, but also with regard to buildings. However, the lack of a 3D presentation domain model of buildings has been identified as a crucial need. A standardized model is therefore essential to achieve a consistent and accurate 3D representation, supported by up-to-date alphanumeric data.

In order for the Ecuadorian cadastre to be able to initiate a path towards the 3D cadastre, it is desired that the components as such be adapted to the general cadastral model proposed in ISO 19152:2012 LADM (2012). It is worth mentioning that the initial proposal put forward by Velastegui *et al*. (2020) considers the spatial unit only up to the parcel; however, in the present research emphasis is placed on buildings with their respective subcomponents to propose a 3D physical cadastral model that corresponds to the 3D representation of buildings with alphanumeric information to be useful in various areas.

The implementation of such a model fills a gap in cadastral management and provides a solid basis for improving urban planning, property rights allocation and decision making in various areas, thus contributing to sustainable development and progress in the Ecuadorian territory.

## 2. Methods

Once the current cadastral model in Ecuador was reviewed, as stated in Ministerial Agreement 017-20 (2020), we proceeded to propose a domain model for 3D representation of buildings for multipurpose purposes in Ecuador, considering both the current model (national standard) and ISO 19152:2012 LADM (international standard) to ensure the applicability of the proposed model and, thus, achieve the transition from 2D to 3D representation of buildings, which in terms of cadastre are considered as physical cadastral objects.

The proposed model is largely based on buildings, allowing their representation in a 3D format. It is not simply a visual representation, but a comprehensive approach that includes alphanumeric information and is fully integrated into the framework of the Ecuadorian cadastral model. In this way, the 3D representation becomes official and authentic information, with varied applications in different areas.

This research develops the domain model for 3D representation of multipurpose buildings in which the initial model proposed by Velastegui *et al*. (2020) is considered. The main purpose of this consideration is to ensure that the proposal can be successfully replicated in various study areas facing similar challenges and constraints. The adaptability and broad applicability of this domain model for 3D representation of buildings are essential to promote significant improvements not only in the engineering area but also in multidisciplinary areas such as cadastral management at national and regional level, and to support sustainable development.

For this purpose, the main focus is on the elements of the physical cadastral object, where the parcel and the building are specified as the spatial unit. Therefore, it is assigned as a subclass to the buildings (EC_BuildingUnit) and in it the information of the structural part that is in the subclass EC_StructuralComponent and the information of the non-structural part in the subclass EC_NoStructuralComponent.

With respect to the coding lists of each of the basic elements, national regulations were considered, as the main guarantor of the standardization of cadastral information throughout the Ecuadorian territory. Specific consideration was given to the category, record number, field, type of data, length/precision and the observations section, in the case of those attributes that had particular characteristics.

In addition, Table 1 presents a synthesis of these associations, with the objective of adapting the international standard ISO 19152:2012 LADM to the specific cadastral reality of the Ecuadorian territory, following the guidelines established by national regulations. This hierarchical structure of relationships between classes supports a more accurate and complete representation of the physical cadastral object (buildings) and the corresponding alphanumeric information, which promotes efficient management of territorial resources and data-driven decision making based on urban planning and territorial development.

**Table 1.** Associations between classes of ISO 19152:2012 LADM (2012).

Class 1	Class 2	association name	Name of the role at end 1	Multiplicity	Name of the role at end 2	Multiplicity
BAUnit (Basic Administrative Unit)	RRR	BAUnitRRR	Unit	1(one)	RRR	1..*(one or many)
Party	BAUnit	BAUnitAsParty	Party	0..*(zero or many)	Unit	0..* (zero or many)
RRR (Right, Restriction and Responsibility)	Party	PartyRRR	RRR	0..* (zero or many)	Unit	0..* (zero or many)
SU (SpatialUnit)	BAUnit	SUBAUnit	SU	0..* (zero or many)	BAUnit	0..* (zero or many)

Source: ISO 19152:2012 LADM (2012).

To implement the proposed domain model for 3D representation of buildings, official cadastral data of the study area were used, which were provided by the Directorate of Appraisal and Cadastre of the Municipality of Riobamba, since they are responsible for preparing and managing urban real estate cadastres within their administrative area.

An area of 13 blocks was selected, located in two sectors, 04 and 05 of the canton, with a total of 261 properties. The selection of the study area was based on the availability and timeliness of data, as well as on the variety of typologies of uses and buildings present in the area. The Department of Appraisal and Cadastre had access to the thematic layers of properties and buildings, which, in their attribute tables, have a field referring to the cadastral key at the parcel level and at the block level, respectively.

We also had access to the paper cadastral files stored on the basis of the cantonal zoning in the archive office of the aforementioned department. The information from the cards, referring to the physical, economic, legal and thematic components, was digitized and incorporated into the attribute table of the thematic layers of properties and buildings, thus completing the cadastral information of the study area.

The usefulness of GIS tools for the generation, updating and management of cadastral information, in general, and of information on certain characteristics of buildings, in particular, is unquestionable. Therefore, Ecuadorian regulations establish that geodatabases must be generated in free software for the storage of this type of data related to parcels and buildings.

In this research, free software was used for its generation. Therefore, the geodatabase was initially developed in pgAdmin 4 ( https://www.pgadmin.org/), which is a graphical interface, developed in Python, essential to manage and administer PostgreSQL ( https://www.postgresql.org/). In order to relate alphanumeric and spatial information and the implementation of the 3D model, QGIS ( https://qgis.org/en/site/) was used, an open source program that has an extension to connect directly with Postgre databases.

## 3. Results

**Figure 1.**
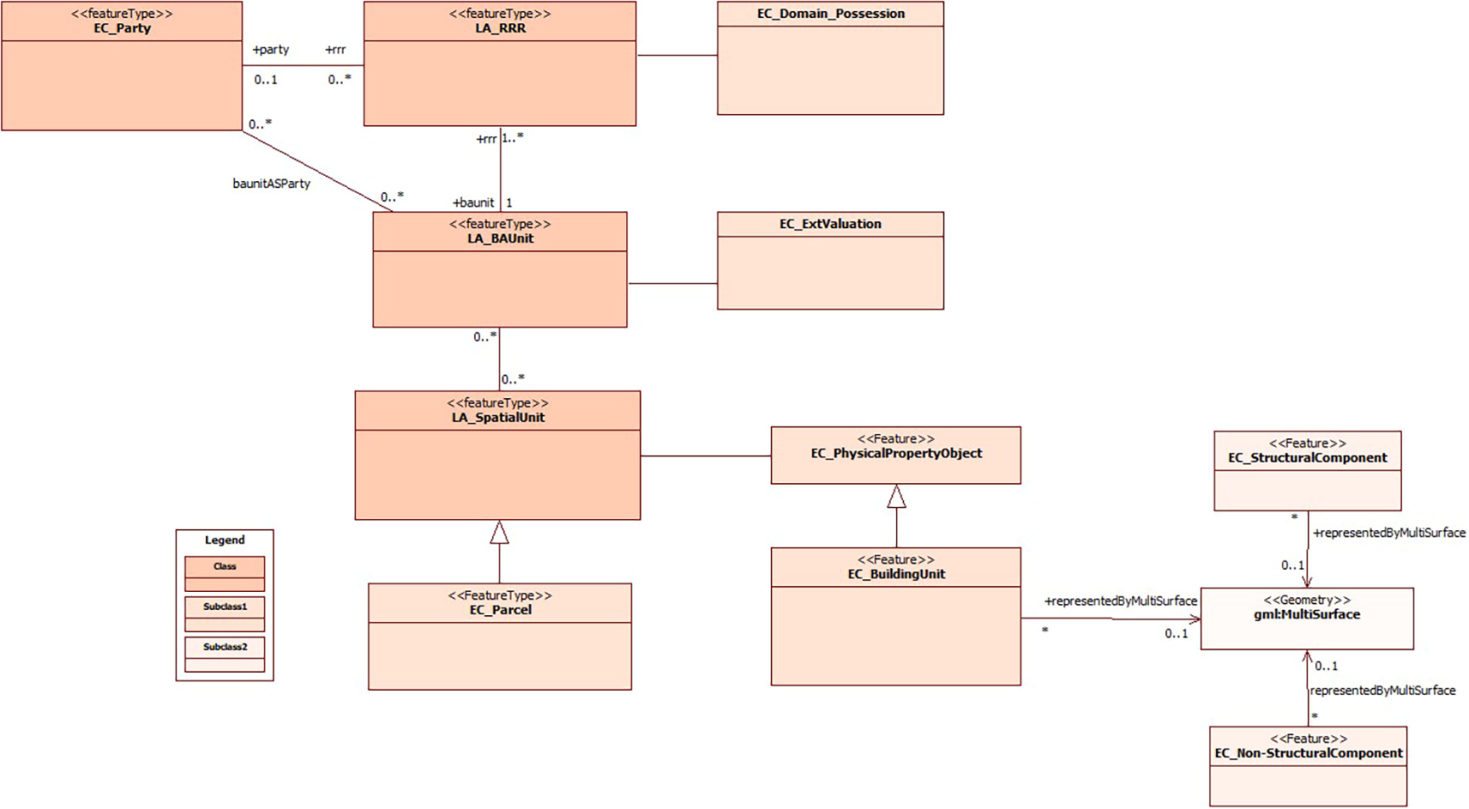
Incorporation of Spatial Unit’s subclasses to the proposed model by Velastegui *et al.* (2020).

Figure 1 provides an overview of the integration of the cadastral model previously proposed by Velastegui *et al.* (2020), with an emphasis on the inclusion of the building unit as an integral part of the spatial unit. This approach allows achieving an accurate 3D representation of the buildings, considering both national regulations and the international standard ISO 19152:2012 LADM.

This process of unification and adaptation to international standards contributes significantly to the standardization of cadastral information management in Ecuador. In addition, it enables a detailed and accurate representation of buildings, which results in better support for urban planning processes, decision making in property matters and the management of territorial resources. The inclusion of the 3D dimension in the cadastre not only improves the visualization and analysis of buildings, but also promotes greater transparency and efficiency in cadastral management, promoting more sustainable urban development and a better quality of life for inhabitants.

### Section EC_PhysicalPropertyObject

The cadastral model presented by Aien *et al*. (2015) offers an innovative approach by integrating legal and physical information in order to achieve more realistic 3D representations in the cadastre. In their model, the physical information is closely related to the legal information and is organized within the PhysicalPropertyObject class.

It is relevant to note that a part of this model has been adopted to represent the sections of the physical component of the Ecuadorian cadastre, specifically regarding buildings, including their structural and non-structural components. This adoption implies an alignment with international standards and best practices, which contributes to a more accurate and consistent representation of buildings in the Ecuadorian cadastre. The incorporation of these innovative approaches in the 3D representation domain model of buildings not only improves the quality of information, but also supports more informed decision making in urban planning and property rights issues.

**Figure 2.**
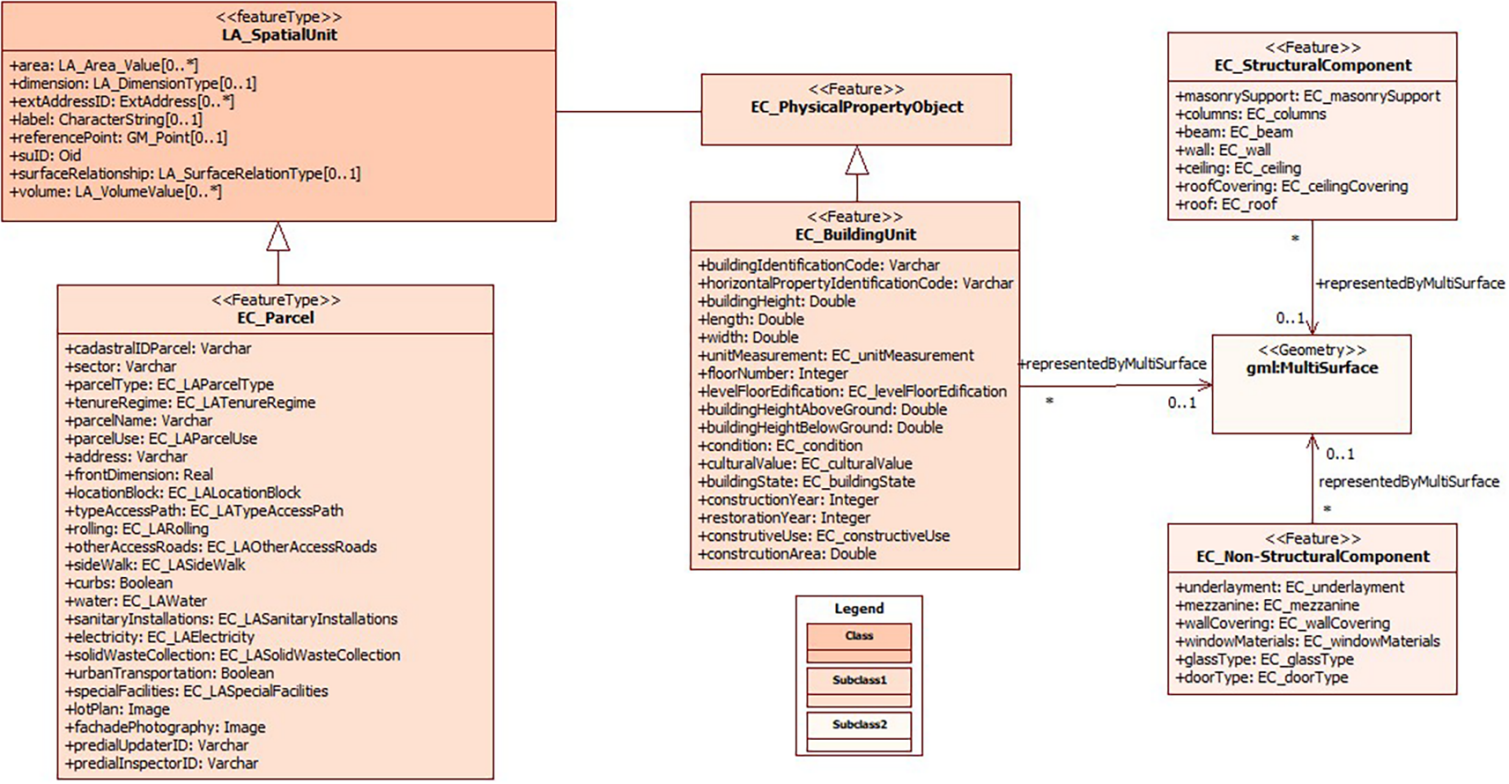
Contents of class EC_PhysicalPropertyObject.

In short, the present model considers, in addition to the legal and physical information of the Ecuadorian regulations, adapting it to ISO 19152:2012 LADM ( Figure 2). Thus, the class LA_SpatialUnit is associated to EC_PhysicalPropertyObject, which contains the physical information of the constructions in EC_BuildingUnit; the information of the structural part of the constructions is found in the subclass EC_StructuralComponent and the information of the non-structural part in the subclass EC_NoStructuralComponent.

Regarding EC_BuildingUnit, there are the main attributes of the building block, where the cadastral key is specified at block, floor and/or unit level and, in turn, the block code to identify the buildings. As part of the attributes, descriptive data of the building block are also considered, such as number of floors, height of the building above and below the parcel (in case there are subway sections that are part of the building such as parking lots, warehouses, among others), existence of construction above and below the parcel, physical condition, state of conservation, patrimonial condition (in case there is a patrimonial value in the construction), year of construction, year of restoration, occupancy of the block or floor.

Regarding the constructive elements of the buildings, specific subclasses were defined for a detailed management of the information. The subclass EC_StructuralComponent focuses on the attributes concerning the structural part of the building, including details on foundations, columns, load-bearing walls and other aspects that are essential from an engineering and structural safety perspective.

On the other hand, the subclass EC_NoStructuralComponent groups and details the attributes related to the non-structural part of the building, which includes elements such as cladding, interior finishes, HVAC systems, windows, doors and other components that do not have a fundamental structural role but are crucial for the functionality and habitability of the building.

The implementation of these subclasses provides a comprehensive approach to the representation and management of the constructive elements of buildings, ensuring that both structural and non-structural aspects are considered in detail in the cadastre. This supports comprehensive decision making in areas such as architectural design, safety, energy efficiency and occupant comfort, which is essential for the quality and sustainability of buildings in the urban context.

The subdivision of information into these subclasses provides a detailed and complete management of cadastral building data, ensuring that all structural and non-structural building elements are accurately represented. The integration of these details into a 3D domain model simplifies the management of cadastral information and enhances informed decision making in matters related to urban planning and land development.

This accurate approach has the potential to optimize the management of urban resources and development policies, while providing decision-makers with a complete and detailed view of the buildings in their environment. The 3D representation of buildings not only contributes to greater efficiency in urban management, but also supports building project planning, structural safety and regulatory compliance, which in turn supports more sustainable and equitable urban development.

**Figure 3.**
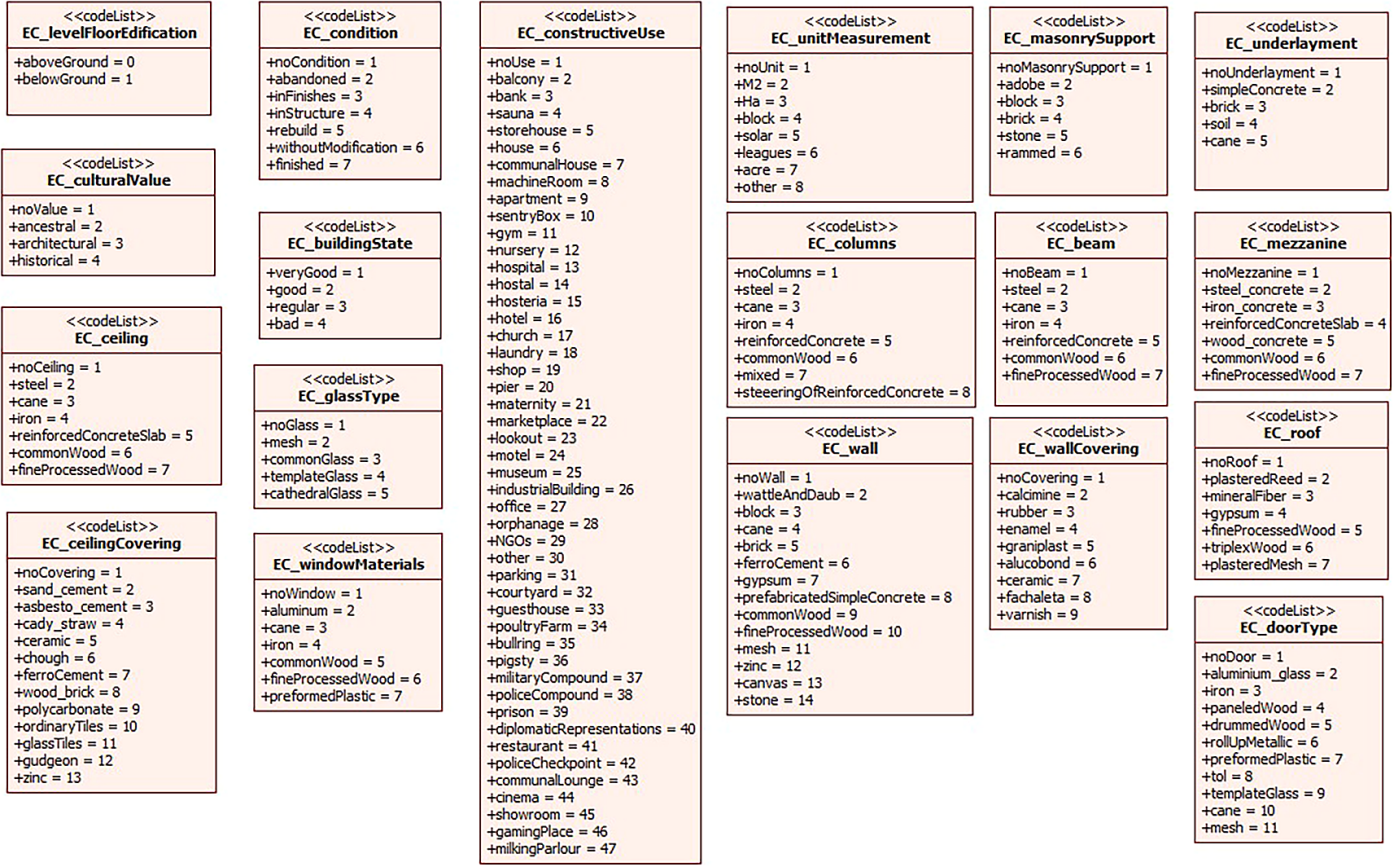
Coding lists of the attributes of the subclasses of EC_PhysicalPropertyObject.

Figure 3 shows the compendium of the coding lists corresponding to the attributes of the subclasses EC_BuildingUnit, EC_StructuralComponent and EC_NoStructuralComponent. It should be noted that the coding lists were developed based on national regulations in order to ensure standardization of the information.

The design process of the domain model for the 3D representation of buildings in Ecuador has followed the structure defined by the ISO 19152:2012 LADM standard. At first, the classes have been established along with their attributes and corresponding list codes, which has provided a solid basis for the 3D representation of the physical cadastral object.

Subsequently, in this specific section, the relationships and associations between the classes proposed in the 3D representation domain model have been addressed. These relationships have been defined considering the order in which the classes are related to each other, which has allowed us to determine the name of the association, the name of the role at end 1 and its multiplicity, as well as the name of the role at end 2 and the corresponding multiplicity.

**Figure 4.**
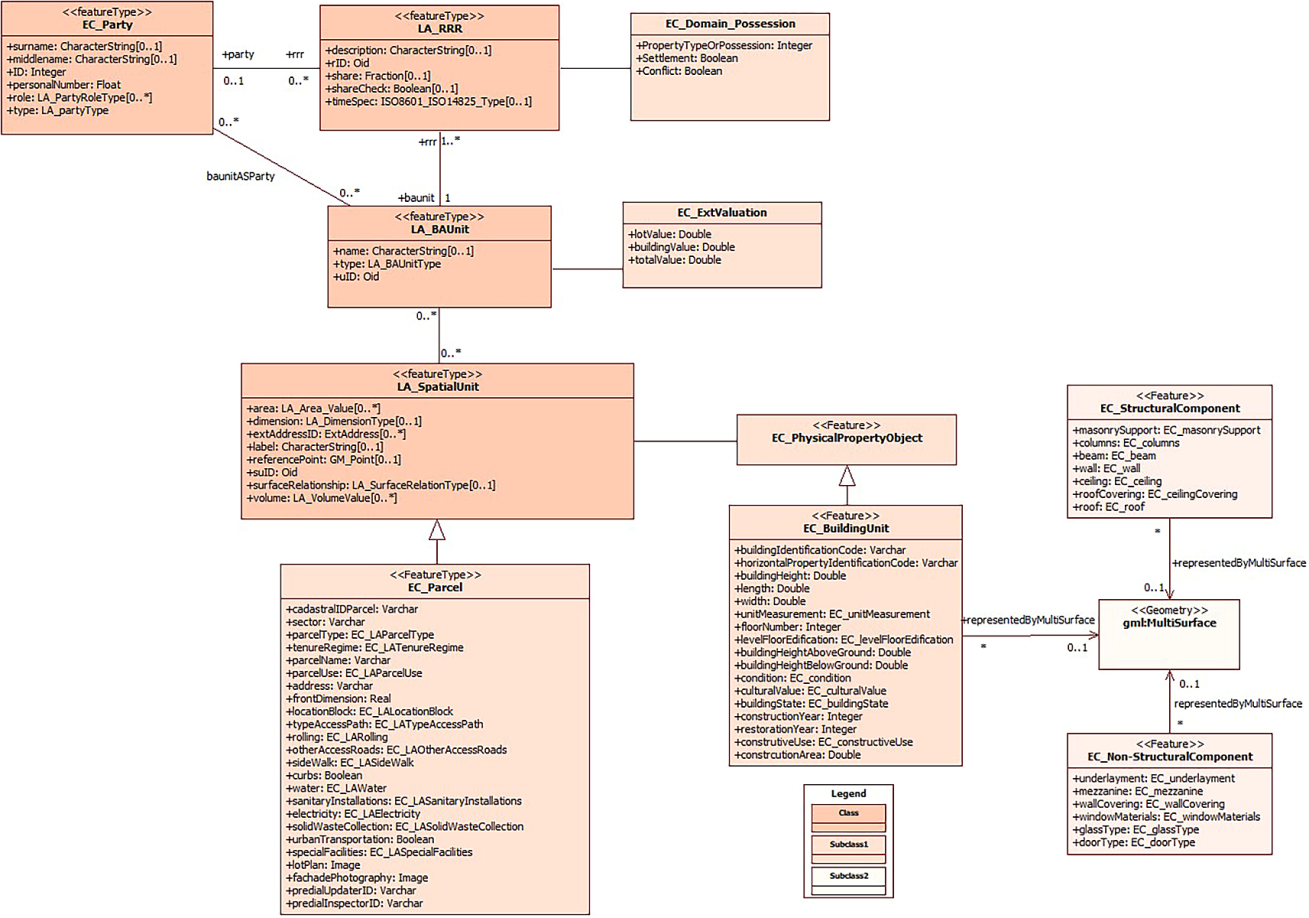
Incorporation of the buildings section to the proposed model by Velastegui *et al.* (2020).

Finally, the proposal of the domain model for the 3D representation of buildings, which aims to lead to the 3D cadastre in Ecuador, is shown in Figure 4. The classes, subclasses and the relationships between them, which are essential to achieve a detailed and complete representation of buildings in three dimensions, are presented here. It is worth noting that the initial model previously proposed by Velastegui *et al*. (2020) served as a starting point for the formulation of this research.

An outstanding feature of this proposal is the inclusion of the building unit component, together with its respective subclasses that address both the structural and non-structural parts of buildings. This approach facilitates the 3D representation of buildings, complemented with alphanumeric information, which is crucial for a variety of multipurpose purposes.

With respect to the implementation of the proposed domain model in a study area of Ecuador, a geodatabase of seven tables was generated that collected alphanumeric data of the administrative unit, physical cadastral object (properties and buildings), owners, rights, responsibilities and restrictions, as well as those associated with the different shapefiles linked to the geodatabase (parcels and buildings).

**Figure 5.**
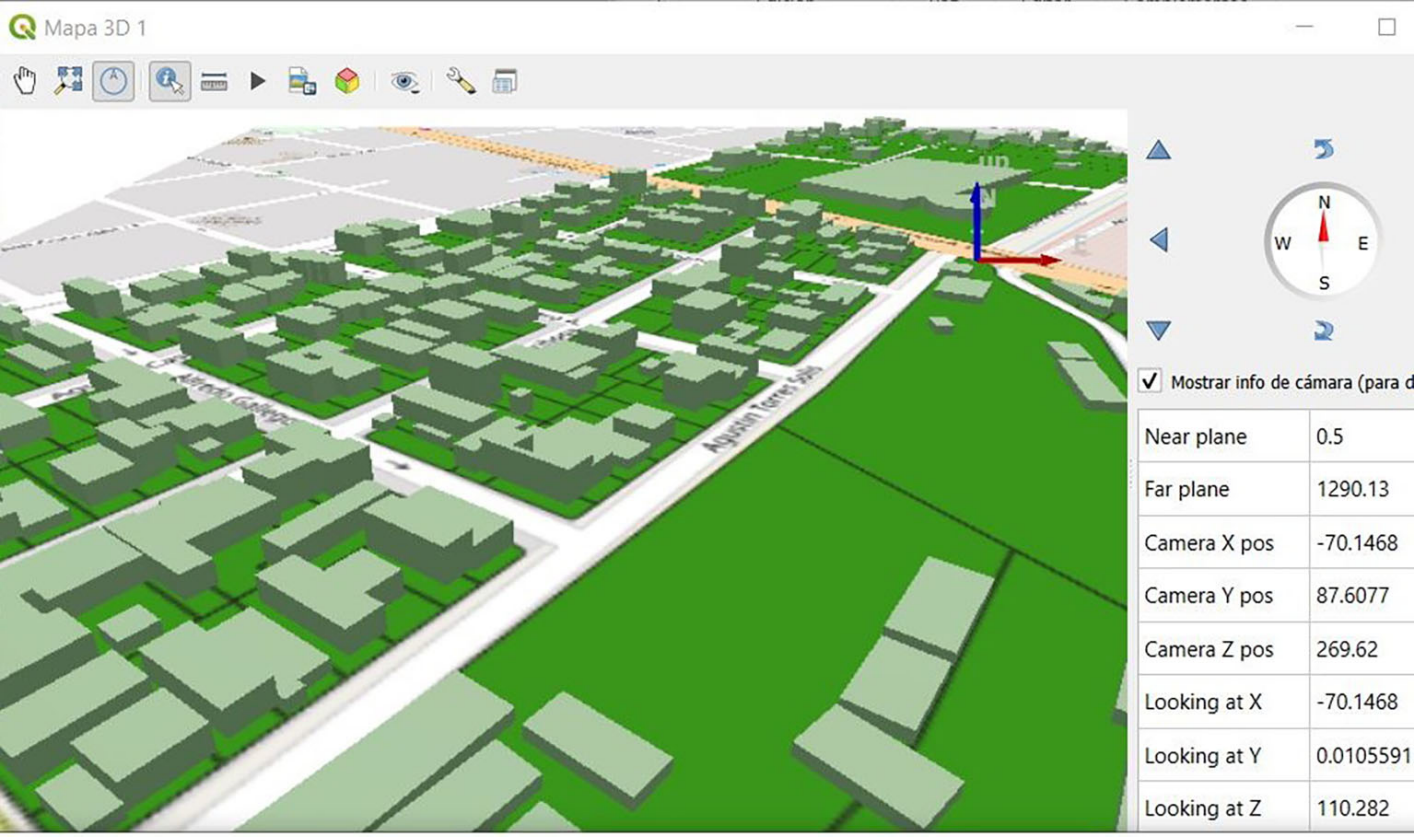
2D and 3D representations of buildings with their respective alphanumeric and geospatial data.

The geodatabase generated and stored in pgAdmin was linked with the parcel and building layers through the PostGIS extension available in the QGIS program; this is a PostgreSQL extension that allows adding geospatial capabilities and functions to ‘conventional’ databases. Once linked, the layers corresponding to the properties and buildings/constructions were added from the QGIS Database Manager. Additionally, the representation of physical cadastral objects at level of detail 1 (LoD1) was obtained based on the information of height and number of floors of the buildings, which allowed an initial 3D modeling ( Figure 5).

This proposed domain model, based on national standards and the international standard ISO 19152:2012 LADM, facilitates a more accurate and complete representation of buildings, which in turn supports urban planning, property rights management and a variety of applications related to territorial development.

## 4. Discussion and conclusions

The proposal of a domain model for 3D representation of buildings could play a fundamental role as a reference point for those developing nations facing similar challenges in terms of their cadastral systems. This initiative would provide them with the opportunity to assess the feasibility of adapting international standards such as ISO 19152:2012 LADM, which would support the first steps towards the implementation of a 3D cadastre. By taking into account the experience of more advanced countries that have successfully implemented this international standard in their transition towards 3D representation of the physical cadastral object ( Zulkifli *et al*., 2017; Janečka & Souček, 2017; Radulović, Sladić & Govedarica, 2017), valuable lessons and best practices could be established to ensure an efficient and effective process.

Adopting an approach based on the experience of more developed nations, in terms of implementing ISO 19152:2012 LADM, could facilitate planning and decision making in countries with fewer resources and technical capacity. This, in turn, would foster greater global consistency and standardization in cadastral information management, promoting the successful transition to 3D cadastre in various regions globally.

The proposed domain model for the three-dimensional (3D) representation of buildings presents a versatility that makes it suitable for replication both nationally and internationally. This is due to the fact that the model is designed with an approach based on national standards, incorporating the international standard ISO 19152:2012 LADM as a fundamental basis. This integration of national and international standards ensures that the proposal is adaptable to the different levels of cadastral development that can be found within the study area.

The ability of this proposal to be implemented in different cadastral contexts, both nationally and internationally, lies in its ability to align with both specific local regulatory requirements and globally supported best practices. This flexibility translates into a powerful tool for the improvement and modernization of cadastral systems in a variety of environments, which, in turn, contributes to the standardization and efficiency of cadastral information management on a global scale.

Considering the spatial unit in parcels and buildings, both spatially and alphanumerically, allows specifying the attributes of each of them, which would facilitate the transition from 2D to 3D physical cadastre. In the case of the building it is possible to consider the general attributes of the building unit and also of the structural and non-structural components, allowing to specify their respective attributes to project in the future an advanced 3D physical cadastre that facilitates cadastral aspects such as the assignment of rights, responsibilities and restrictions at the level of actors, both municipal and owners.

The implementation of the domain proposal for the 3D representation of buildings involves an essential process that begins with the acquisition of official data, most of which is provided by municipalities. This step is crucial to ensure that the data meets the minimum requirements stipulated by national regulations. Through the collection of reliable and accurate information, the necessary basis is laid for the creation of a geodatabase that allows proper processing, management and 3D representation of the physical cadastral objects.

The use of official data and compliance with national regulations are key elements to ensure the integrity and quality of the spatial unit (parcel/building) information in the 3D environment. This, in turn, facilitates more efficient and accurate database management for 3D representation, which is essential for urban planning, property rights allocation, regulatory compliance and informed decision making. Collaboration between municipalities and government entities responsible for cadastral regulation is essential to guarantee the effectiveness of this process and to ensure the availability of reliable and up-to-date data for the benefit of society at large.

In order to successfully implement the domain model for the 3D representation of buildings, it is essential to have data that meet certain key criteria. Firstly, it is strongly recommended that the data used has been updated within a period of no more than two years. This ensures that the information is as accurate and relevant as possible, which is essential for an accurate 3D representation.

In addition, it is essential that the data have a unique identifier, such as a cadastral key, at all levels, whether parcel or building. This allows the effective linking of geospatial and alphanumeric data, which facilitates the management and consultation of information related to buildings.

Finally, the data must be presented in formats compatible with the software to be used in the process, ensuring that the data can be easily integrated into the information system and processed efficiently.

These criteria contribute greatly to the quality and efficiency of the process of implementing the 3D domain model representation of buildings, enabling accurate representation and facilitating the effective management of cadastral information in a unified system. The availability of up-to-date and compatible data is essential for effective decision making in urban planning and cadastral management.

Finally, it can be noted that the implementation of a domain model for the 3D representation of buildings plays a vital role not only in the field of engineering but also in multidisciplinary areas. This revolutionary approach allows for a more accurate and complete representation of structures, which translates into more effective planning and design of construction projects. The ability to visualize and analyze buildings in three-dimensional space provides a deeper understanding of the challenges and opportunities in each project. In addition, detailed 3D building information facilitates more efficient management of resources and time, resulting in significant cost savings and reduced risks associated with construction. Ultimately, the implementation of a 3D domain model has become a fundamental pillar for the execution of more accurate, safe and sustainable projects.

## Data Availability

No data are associated with this article. Zenodo: Proposal of a domain model for 3D representation of buildings for the 3D cadastre in Ecuador, https://10.5281/zenodo.10403713 ( Velastegui Cáceres *et al*., 2023). Data are available under the terms of the Creative Commons Attribution 4.0 International license (CC-BY 4.0). The cadastral data for this project have been obtained from: https://georiobamba-gadmriobamba.hub.arcgis.com/

## Software availability

This project was made possible thanks to the following open source software:

• pgAdmin 4 ( https://www.pgadmin.org/);
• PostgreSQL ( https://www.postgresql.org/);
• QGIS ( https://qgis.org/en/site/).
