# Supplementary material for: Proposal of a domain model for 3D representation of buildings in Ecuador
Source: F1000Res. 2024 Jun 28;13:301. Originally published 2024 Apr 19. [Version 2] doi: 10.12688/f1000research.146267.2 (PMC11217719; doi:10.12688/f1000research.146267.2)
Supplement: Peer review history

# Reviewer report: Reviewer response for version 2

**DOI:** 10.5256/f1000research.168298.r296931

**Published:** 2024-07-01

**Authors:** Uznir Ujang

**Affiliations:** 1 Universiti Teknologi Malaysia, Johor, Malaysia

The authors have addressed my previous comments comprehensively, providing justifications and making the necessary revisions to improve the quality of the paper.

Is the case presented with sufficient detail to be useful for teaching or other practitioners?

Partly

Is the work clearly and accurately presented and does it cite the current literature?

Partly

If applicable, is the statistical analysis and its interpretation appropriate?

Not applicable

Are all the source data underlying the results available to ensure full reproducibility?

Partly

Are the conclusions drawn adequately supported by the results?

Partly

Is the background of the case’s history and progression described in sufficient detail?

Partly

Reviewer Expertise:

3D City Modeling, 3D GIS, Topology, Geo-BIM, GIS

I confirm that I have read this submission and believe that I have an appropriate level of expertise to confirm that it is of an acceptable scientific standard.

# Reviewer report: Reviewer response for version 1

**DOI:** 10.5256/f1000research.160331.r285537

**Published:** 2024-06-19

**Authors:** John Samuel

**Affiliations:** 1 Enseignant-Chercheur, CPE Lyon, Lyon, France

The case study "Proposal of a Domain Model for 3D Representation of Buildings in Ecuador" presents a model for 3D representation of buildings, considering the regulations in Ecuador. The study, spanning four sections, is presented in a well-structured manner.

 Section 1 provides the necessary background for the proposed study, presenting some existing works related to the representation of cadastral information, with a particular focus on 3D information. Additionally, this section presents the context of Ecuador, both in terms of the desired functionalities and the current regulations and limitations.

 However, I have a significant remark for this section, which I believe can further improve the article. The authors state that "The implementation of a 3D cadastre is not a simple task, as it involves numerous challenges as demonstrated by the researches implemented so far," followed by a list of citations. It is unclear what challenges are presented in this extensive list of articles. I believe the authors could highlight some of the key challenges with references to one (or more, if necessary) articles from this list.

 Section 2 presents the methods, considering the Ministerial Agreement (2020) and basing their proposed method on previous work by Velastegui et al. (2020). They present the selected international standards and explain the open-source tools used in this study.

 In this section (as well as the previous section), I suggest the authors elaborate more on the study area and its limitations. They state that “The physical 3D model considers the limitations of the study area to be replicated in areas with characteristics similar to Ecuador”. While readers may infer some limitations from the article, these should be clearly presented in Section 1 or 2.

 Section 3 presents the results, especially the proposed extensions to the models and the 3D visualization of a selected area in QGIS with an extension.

 In this section, my major concern is with Figures 4 and 5. Although the authors have clearly stated that they have extended the previous work of Velastegui et al. (2020), it would be helpful if they highlighted their newly proposed contributions in Figure 4 with different colors or textures. Regarding Figure 5, the authors mention an extension of PostGIS for the 3D visualization, but they need to describe the extension (presumably Mapa 3D, based on the screenshot in Figure 5).

 Finally, Section 4 concludes the article, discussing their proposed models and possible future work. The work is promising, especially because it demonstrates how they consider the local context while remaining global by using international standards.

 Overall, the case study is well-written. However, there is room for improvement. The authors need to consider international standards like CityGML, which already proposes 3D structural information of buildings and some information related to non-structural components. Some proposed attributes could be further improved or justified by comparing them with such city models.

Is the case presented with sufficient detail to be useful for teaching or other practitioners?

Yes

Is the work clearly and accurately presented and does it cite the current literature?

Partly

If applicable, is the statistical analysis and its interpretation appropriate?

Not applicable

Are all the source data underlying the results available to ensure full reproducibility?

Partly

Are the conclusions drawn adequately supported by the results?

Yes

Is the background of the case’s history and progression described in sufficient detail?

Yes

Reviewer Expertise:

Data Integration, Data Warehouses, Data Analysis, Big Data, Web Services, Knowledge Representation, Semantic Web, Multidimensional Data Modeling, Description Logic, Geographical Information Systems

I confirm that I have read this submission and believe that I have an appropriate level of expertise to confirm that it is of an acceptable scientific standard.

# Response

**Published:** 2024-06-23

**Authors:** Pedro Antonio Carretero Poblete

**Affiliations:** Facultad de Ingeniería, Universidad Nacional de Chimborazo, Riobamba, Chimborazo Province, Ecuador

Dear Dr. John Samuel,

 Thank you very much for your valuable comments and suggestions on our manuscript. We have carefully considered your comments and made the necessary revisions in the new version of the article.

# Reviewer report: Reviewer response for version 1

**DOI:** 10.5256/f1000research.160331.r274274

**Published:** 2024-05-29

**Authors:** Uznir Ujang

**Affiliations:** 1 Universiti Teknologi Malaysia, Johor, Malaysia

The research article presents a well-defined problem and rationale for proposing a 3D representation model for buildings in Ecuador. The need for detailed 3D building models for urban planning and resource management is articulated clearly. However, further clarification on the current limitations of the existing 2D cadastre and the specific challenges it poses in the Ecuadorian context would strengthen the introduction.

 1.    Page 3 - How does the proposed model address specific challenges unique to Ecuador that might not be present in other countries with similar urban development issues?

 2.    Page 4 - What are the key differences between the national standard of Ecuador and the international standard ISO 19152:2012 LADM that necessitated the specific adaptations you made?

 3.    How do you ensure the proposed model's scalability and applicability to other regions in Ecuador or other countries with different cadastral development stages?

 4.    Can you provide more detail on the validation process of the proposed model? How was it tested, and what were the criteria for its success?

 5.    How do you plan to address the potential lack of technological infrastructure and expertise in some regions of Ecuador for implementing the 3D cadastre?

 6.    What were the specific criteria used to select the 13 blocks in the study area, and how do these criteria ensure the representativeness of the study?

 7.    How does the proposed 3D model integrate with existing cadastral systems and databases currently used by Ecuadorian municipalities?

 8.    What measures are in place to ensure the continuous updating and accuracy of the data within the proposed 3D cadastral model?

 9.    How does the model handle buildings with complex structures, such as those with multiple sub-levels or mixed-use purposes?

 Suggestions for Improvement:

 1.    The literature review is comprehensive but can benefit from a deeper analysis of case studies where 3D cadastral models have been successfully implemented. Highlighting the outcomes and lessons learned from these implementations can provide more context and justification for your model.

 2.    The methodology section is detailed but can be improved by providing a step-by-step outline of the process used to develop the geodatabase and the tools employed. Including diagrams or flowcharts to illustrate the methodology would enhance clarity and comprehension.

 3.    Incorporate more detailed examples of the 13 blocks selected for the study, including before and after comparisons of 2D and 3D representations. This will provide concrete evidence of the model's effectiveness.

 4.    Discuss the potential challenges and limitations encountered during the implementation of the proposed model, such as data quality issues, integration difficulties, and any resistance from local authorities or stakeholders.

 5.    Outline the next steps for the broader implementation of the 3D cadastral model in Ecuador. Discuss plans for scaling the model to other regions and how you intend to handle potential scalability issues.

Is the case presented with sufficient detail to be useful for teaching or other practitioners?

Partly

Is the work clearly and accurately presented and does it cite the current literature?

Partly

If applicable, is the statistical analysis and its interpretation appropriate?

Not applicable

Are all the source data underlying the results available to ensure full reproducibility?

Partly

Are the conclusions drawn adequately supported by the results?

Partly

Is the background of the case’s history and progression described in sufficient detail?

Partly

Reviewer Expertise:

3D City Modeling, 3D GIS, Topology, Geo-BIM, GIS

I confirm that I have read this submission and believe that I have an appropriate level of expertise to confirm that it is of an acceptable scientific standard, however I have significant reservations, as outlined above.

# Response

**Published:** 2024-06-23

**Authors:** Pedro Antonio Carretero Poblete

**Affiliations:** Facultad de Ingeniería, Universidad Nacional de Chimborazo, Riobamba, Chimborazo Province, Ecuador

Dear Dr. Uznir Ujang,

 Thank you for your valuable comments and suggestions on our manuscript. We appreciate the time and effort you have dedicated to providing feedback that will help improve the quality of our work.

 In replying to your comments, we took your comments into consideration for the new version of the article to provide a greater understanding of the limitations and challenges in transitioning from a 2D cadastre to a 3D cadastre.

1. In Ecuador, there are regulations for the generation of cartographic inputs, standardization of databases, and catalogs of objects at different scales. Despite this, various procedures for the collection, storage, processing, and visualization of building-related data exist in the country. This diversity limits the adequate representation of buildings, resulting in the predominance of 2D representation of buildings in the country. Proper organization and interoperability of building-related information are fundamental to initiating 3D representations. The proposed domain model considers both national and international standards to more accurately characterize the set of existing buildings.
2. The main difference starts from its general structure. The national standard considers four components: economic, physical, legal, and thematic. On the other hand, the international standard consists of party, basic administrative unit, RRR (rights, restrictions, and responsibilities), and spatial unit. The structure presented by the international standard tends to be more flexible and adaptable to different scenarios. Considering that Velastegui et al. (2020) propose adapting and merging the general structure of the national standard with the international one, this model is used to specifically address the Spatial Unit (parcel and building). The adapted Spatial Unit class essentially considered the minimum elements established by the national standard. While it does not limit these to being the only elements, it leaves room for the incorporation of additional elements. To incorporate necessary elements into the spatial unit (building unit), the international standard was taken into account, thus adapting structural and non-structural components to the Building Unit subclass.
3. To ensure that the proposed model can be applied in different areas of the country, the cadastral development levels of the country were considered. Based on this, a model is proposed that covers the most predominant characteristics of the different areas. For application, a study area representing these characteristics was considered to verify the adaptability of the proposed model. However, as next steps, pilot projects are planned in other cities or regions according to their population density, urban development, and specific needs to validate and adjust the model in different contexts. Based on the pilot projects, performance indicators will be established to monitor the progress and effectiveness of the implementation.
4. Workshops were organized with cadastre technicians, particularly those in charge of geospatial data, to obtain direct feedback on the usability and functionality of the 3D model. As a result of the suggestions and observations from end users, the necessary adjustments and improvements were made to the model. The criteria considered interoperability and usability to ensure that the model can be integrated with various cadastral systems and databases, as well as adopted and used effectively by users.
5. Technological infrastructure challenges are a concern not only for the implementation of the 3D cadastre but for cadastres at all levels. Therefore, the Ministry of Urban Development and Housing, through Agreement 017-20, sets forth transitional provisions indicating that municipalities must standardize and adapt data to a Local Cadastral Geographic Information System (SIGCAL). For this, SIGCAL must meet a series of essential requirements such as being open-source; having an integrated database; allowing implementation on different operating systems and access through various web browsers; and having interoperability capabilities and web-oriented or desktop architecture.
6. The selection criteria for the study area were mainly based on the availability and updating of both geospatial and alphanumeric data. Additionally, the level of cadastral development was considered, as a high percentage of areas in the country have intermediate cadastral development with challenges similar to the selected area. Blocks containing the most representative and diverse characteristics of the study area were defined, such as building types, year of construction, data updates, construction diversity, land use, etc. Therefore, the implementation of the domain model proposal was carried out in 13 blocks that met the mentioned criteria.
7. The integration of the initial 3D model proposal was challenging primarily due to interoperability issues. Databases were used where the geospatial and alphanumeric components were not fully integrated. Therefore, it was necessary to verify the compatibility of both alphanumeric and geospatial data formats. However, with the model proposal, it was feasible to implement free, integrated databases that allowed the storage of both geospatial and alphanumeric components associated through the cadastral key, which is the unique identifier of the cadastral object (parcel/building). The requirement of Ministerial Agreement 017-20 was met by using open-source GIS software that guarantees interconnection with previously implemented platforms and systems. Socializing the process with end users is fundamental to establishing protocols at different levels of cadastral systems.
8. The Ministry of Urban Development and Housing, through the Ministerial Agreement issued in 2020, presents transitional provisions directed at all municipalities in the country to ensure that, regardless of their cadastral development, they adhere to these provisions. Each municipality must update the urban cadastre considering guidelines to ensure data accuracy and quality. These transitional provisions have an established timeframe for compliance, thus ensuring more uniform levels of cadastral development across different areas of the country.
9. The majority of buildings in Ecuador meet the characteristics considered by this domain model. The present model is an initial proposal; however, future research on buildings with complex structures is planned.

The authors appreciate your suggestions for improvement, which have been incorporated into the new version of the article as detailed below:

1. An analysis of case studies where 3D cadastral models were successfully implemented has been incorporated into the introduction section.
2. A diagram regarding the development of the geodatabase has been added to the methods section.
3. Additional information regarding the 13 blocks selected for the study, as well as, comparisons of before and after 2D and 3D representations have been included in the results section.
4. Possible challenges and limitations encountered during the implementation of the proposed model have been added to the discussion and conclusions section.
5. Future steps for the broader implementation of the 3D cadastral model in Ecuador and plans to scale the model to other regions have been added to the discussion and conclusions section.
